# Identification of a Novel Heterozygous *De Novo* 7-bp Frameshift Deletion in *PBX1* by Whole-Exome Sequencing Causing a Multi-Organ Syndrome Including Bilateral Dysplastic Kidneys and Hypoplastic Clavicles

**DOI:** 10.3389/fped.2017.00251

**Published:** 2017-11-24

**Authors:** Korbinian Maria Riedhammer, Corinna Siegel, Bader Alhaddad, Carmen Montoya, Reka Kovacs-Nagy, Matias Wagner, Thomas Meitinger, Julia Hoefele

**Affiliations:** ^1^Department of Nephrology, Klinikum rechts der Isar, Technical University of Munich, Munich, Germany; ^2^Institute of Human Genetics, Klinikum rechts der Isar, Technical University of Munich, Munich, Germany; ^3^KfH Center of Pediatric Nephrology, Children’s Hospital Munich Schwabing, Munich, Germany; ^4^Institute of Human Genetics, Helmholtz Zentrum Munich, Neuherberg, Germany; ^5^Institute of Neurogenomics, Helmholtz Zentrum Munich, Neuherberg, Germany

**Keywords:** CAKUT, *PBX1*, dysplastic kidneys, hypoplastic clavicles, developmental delay

## Abstract

**Introduction:**

Congenital anomalies of the kidney and urinary tract (CAKUT) represent the primary cause of chronic kidney disease in children. Many genes have been attributed to the genesis of this disorder. Recently, haploinsufficiency of *PBX1* caused by microdeletions has been shown to result in bilateral renal hypoplasia and other organ malformations.

**Materials and methods:**

Here, we report on a 14-year-old male patient with congenital bilateral dysplastic kidneys, cryptorchidism, hypoplastic clavicles, developmental delay, impaired intelligence, and minor dysmorphic features. Presuming a syndromic origin, we performed SNP array analysis to scan for large copy number variations (CNVs) followed by whole-exome sequencing (WES). Sanger sequencing was done to confirm the variant’s *de novo* status.

**Results:**

SNP array analysis did not reveal any microdeletions or -duplications larger than 50 or 100 kb, respectively. WES identified a novel heterozygous 7-bp frameshift deletion in *PBX1* (c.413_419del, p.Gly138Valfs*40) resulting in a loss-of-function. The *de novo* status could be confirmed by Sanger sequencing.

**Discussion:**

By WES, we identified a novel heterozygous *de novo* 7-bp frameshift deletion in *PBX1*. Our findings expand the spectrum of causative variants in *PBX1*-related CAKUT. In this case, WES proved to be the apt technique to detect the variant responsible for the patient’s phenotype, as single gene testing is not feasible given the multitude of genes involved in CAKUT and SNP array analysis misses rare single-nucleotide variants and small Indels.

## Introduction

Congenital anomalies of the kidney and urinary tract (CAKUT) represent the primary cause of chronic kidney disease in children. CAKUT is the collective term for many different renal and urinary tract malformations. In recent years, a multitude of monogenic disease-causing genes has been discovered (1). Disruption of the normal nephrogenesis by pathogenic variants in genes involved in kidney development is a basic principle of CAKUT (2).

When it comes to heterogeneous diseases like CAKUT, with many different genes involved, large-scale next-generation sequencing has become an extremely useful tool for the unbiased detection of pathogenic variants (3). In whole-exome sequencing (WES), the coding regions (the exome) of the human genome are enriched and sequenced. This has proven to be both an economic, as the exome only comprises 1% of the genome, and a pragmatic approach, as about 85% of disease-related variants can be found in the exome (4).

*PBX1* encodes a transcription factor that has already been linked to nephrogenesis as shown by *Pbx1*-deficient mice (5). Additionally, earlier mouse models revealed its role in bone formation (6). In 2017, microdeletions comprising *PBX1* as a minimal common region could be identified by microarray analysis in eight patients with syndromic CAKUT with predominantly renal hypoplasia. In the same publication, it was shown that *PBX1* was strongly expressed in the fetal kidney and brain (7). Here, we report on a 14-year-old male patient presenting to the pediatric nephrology department with the predominant clinical features of bilateral dysplastic kidneys, hypoplastic clavicles, and developmental delay.

## Materials and Methods

This study was approved by the local Ethics Committee of the Technical University of Munich and performed according to the standard of the Helsinki Declaration of 1975. Written informed consent was obtained from the parents of the participant for publication of this case report. Blood samples were collected after written informed consent.

DNA was extracted from peripheral blood using the Gentra Puregene Blood Kit (Qiagen, Hilden, Germany) according to the manufacturer’s instructions. The DNA sample of the patient was analyzed by using the SNP Array Affymetrix^®^ CytoScanTM 750 K Array (Affymetrix^®^ Inc., Santa Clara, CA, USA) with an average space between two oligonucleotides of 4 kb. Scanning was performed by the Affymetrix^®^ GeneChip Scanner 3000 7G (resolution 0.51–2.5 µm). The data analysis was conducted using the Affymetrix^®^ Chromosome Analysis Suite Software (ChAS), version 3.0, hg19.

Exome sequencing was performed using a Sure Select Human All Exon 60 Mb V6 Kit (Agilent) and a HiSeq4000 (Illumina) as previously described (8, 9). Reads were aligned to the UCSC human reference assembly (hg19) with BWA v.0.5.8. More than 98% of the exome was covered at least 20×. *PBX1* was covered >20× to 100%. Single-nucleotide variants and small insertions and deletions were detected with SAMtools v.0.1.7. Variant prioritization was performed based on an autosomal recessive pattern of inheritance (homozygous or putative compound heterozygous variants with a minor allele frequency <0.1%) as well as an autosomal dominant pattern of inheritance (heterozygous variants with a minor allele frequency <0.001%).

Sanger sequencing was used to confirm the identified variant and to test the patient’s parents. Oligonucleotide primer sequences are available upon request.

## Case Report and Results

**Figure 1:**
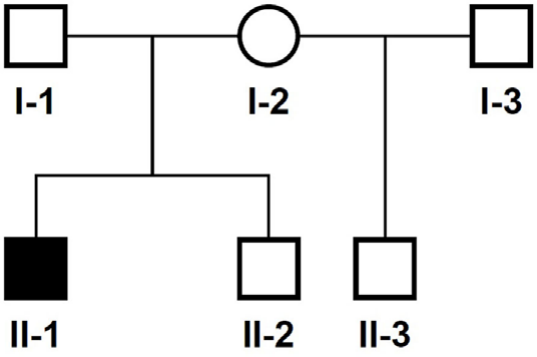
Pedigree of the family. Solid symbol, affected individual; circles, females; squares, males.

**Figure 2:**
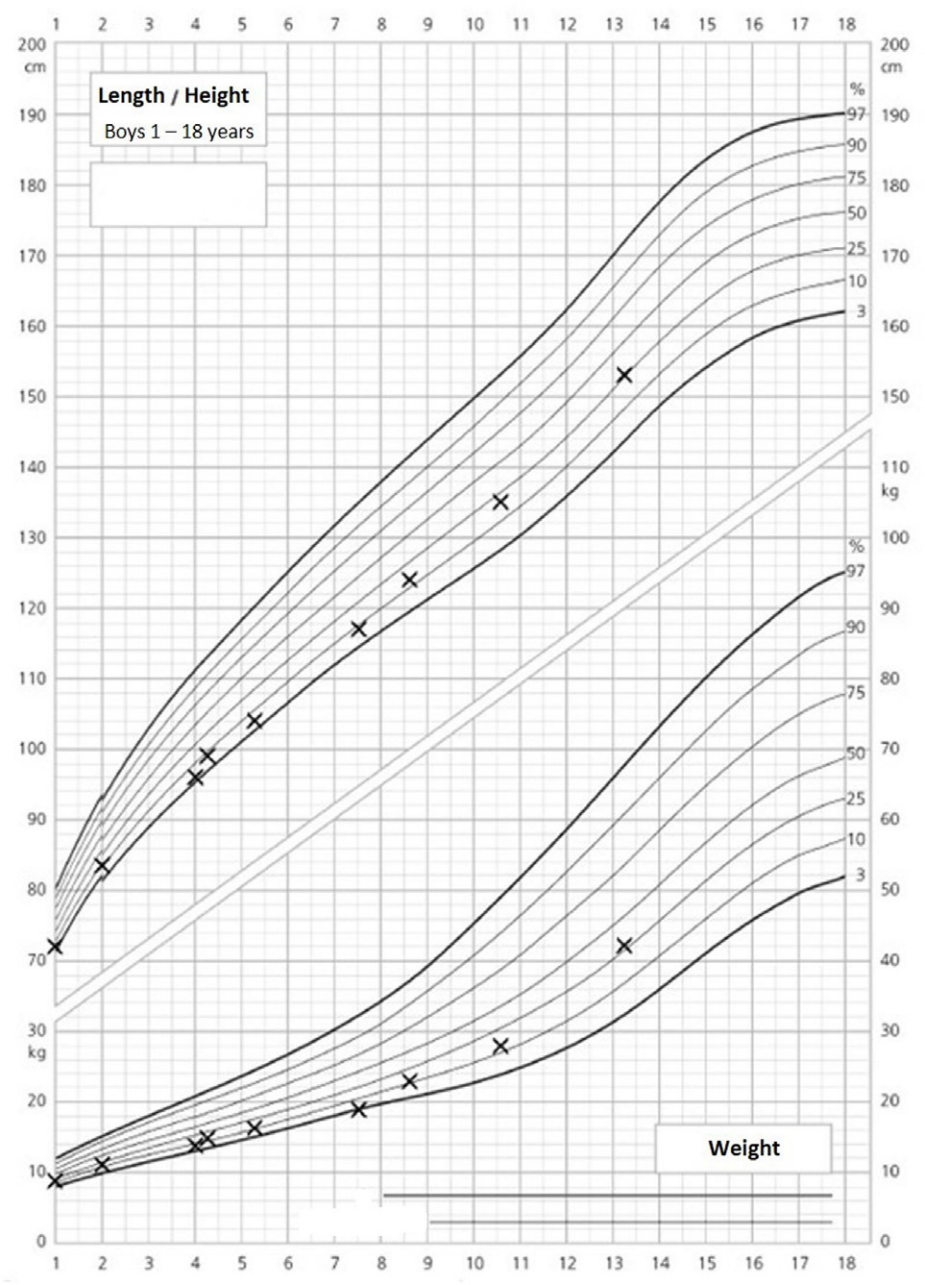
Growth and weight charts of the patient. These charts are gender specific and refer to boys. *x*-axis, age (years); *y*-axis, height (centimeter, upper part of the figure) and weight (kilograms, lower part of the figure).

The 14-year-old boy is the first child of healthy parents (Figure 1). He has one younger healthy brother and one younger healthy half-brother. The patient was born at a gestational age of 38 weeks [birth weight: 2,840 g (25th–50th percentile), birth length: 48 cm (10th–25th percentile)]. There were no obvious malformations noted at birth. During the neonatal period, a slender thorax and short clavicles were identified clinically. Body height was on the third percentile during infancy and early childhood (Figure 2) and a global developmental delay (including motor and speech delay) was diagnosed on regular pediatric screening examinations.

**Figure 3:**
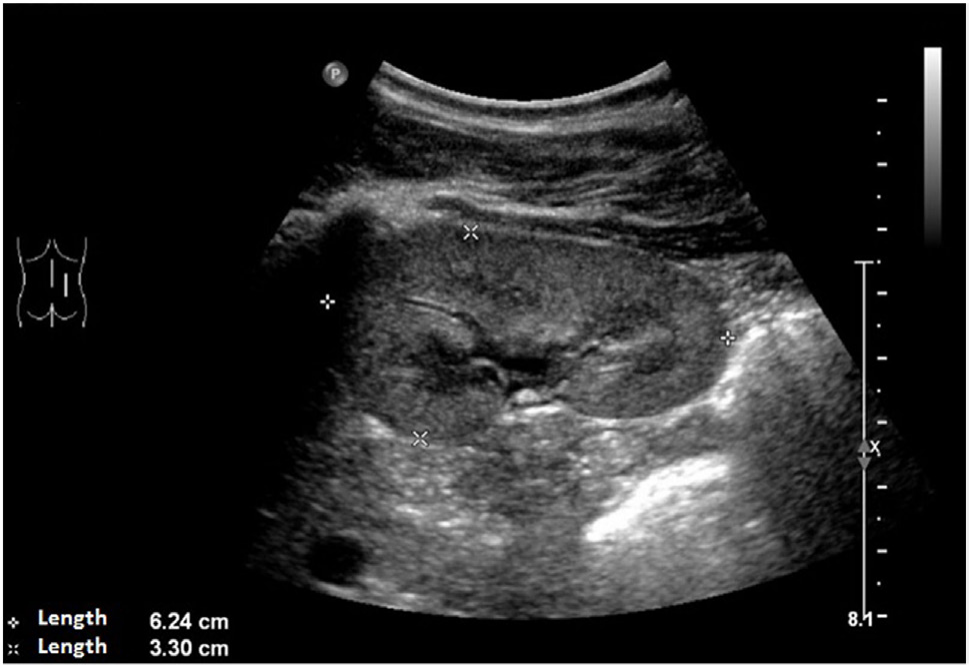
Ultrasound of the right kidney with a length of 6.2 cm (<1st percentile).

**Figure 4:**
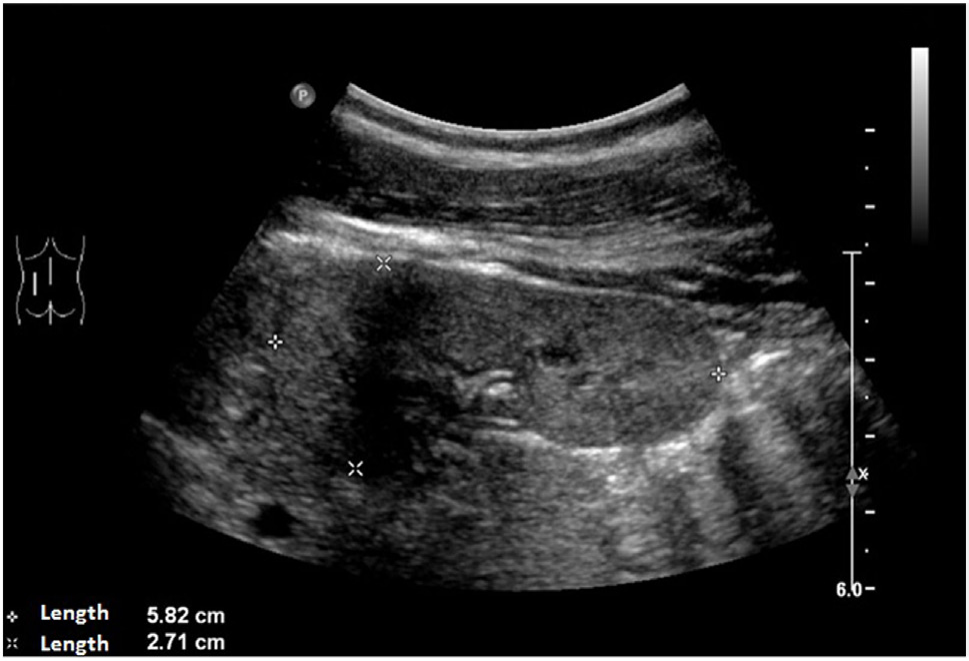
Ultrasound of the left kidney with a length of 5.8 cm (<1st percentile).

An ultrasound of the kidneys at 3 months of age revealed small kidneys with hyperechogenic parenchyma. The boy was then regularly seen by a pediatric nephrologist. By the age of six, the right kidney had a length of 6.2 cm (<1st percentile, see Figure 3), the left kidney had a length of 5.8 cm (<1st percentile, see Figure 4). Kidney function ranged between eGFR 59 mL/min/1.73 m^2^ at 4 years of age, 90 mL/min/1.73 m^2^ at 8 years of age, and 69 mL/min/1.73 m^2^ at 13 years of age (Schwartz estimate, CKD II). Moreover, the patient had bilateral cryptorchidism for which he received hormonal therapy by the age of 2 years.

Psychological intelligence testing by the age of 13 revealed a below average speech comprehension (IQ 81), a reduced information processing speed (IQ 74) and an impaired auditory working memory (IQ 77).

**Figure 5:**
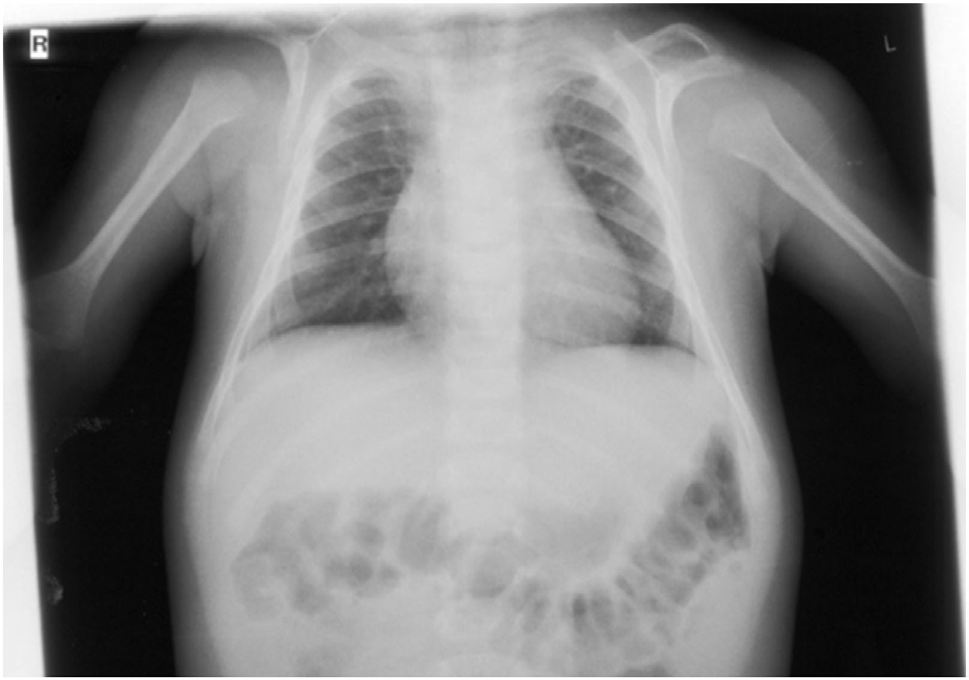
X-ray (detail of a babygram) of the patient at the age of 7 months showing a hook-shaped hypoplastic left clavicle.

On orthopedic examination the patient had a slim shoulder profile, impaired abduction of the arms, and a hunched back. Thoracic X-ray revealed hypoplastic clavicles (Figure 5, only left clavicle visible). He also had some dysmorphic features (wide nasal bridge, short neck, bilateral overfolding of the helix, and bilateral clinodactyly).

**Figure 6:**
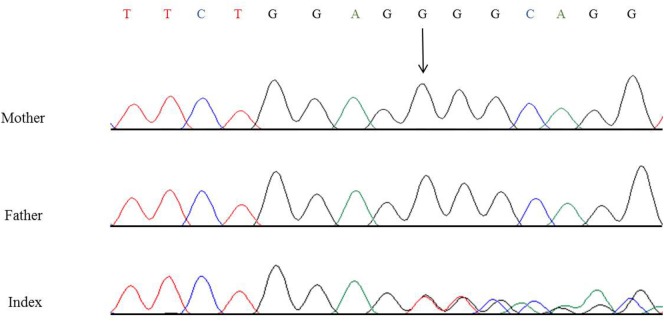
Partial nucleotide sequence of exon 3 of *PBX1* of the patient and his parents showing the heterozygous *de novo* variant c.413_419delGGGCAGG, p.Gly138Valfs*40. Shown reference sequence: TTCTGGAGGGGCAGG. Genomic position of the variant: chr1:164761876–164761882 (hg19, transcript NM_002585.3).

In this child, we presumed a syndromic origin and initially performed SNP array analysis. However, no microdeletions or duplications (copy number variations, CNVs) larger than 50 or 100 kb, respectively, could be detected. As CAKUT have been associated with a large number of genes, we then performed WES. By WES, we could identify a novel heterozygous 7-bp deletion in *PBX1* leading to a frameshift (c.413_419delGGGCAGG, p.Gly138Valfs*40), resulting in either nonsense-mediated decay of the mRNA or a truncated protein lacking the DNA-binding domain. The variant is not listed in 60,000 control individuals of the Exome Aggregation Consortium (ExAC) browser. The ExAC browser does not list any high-confidence loss-of-function variants in *PBX1* indicating that *PBX1* is intolerant to loss-of-function variants. To verify the *de novo* status of the variant, we performed Sanger sequencing in the patient and his parents. The variant could not be detected in the blood DNA of both parents (Figure 6).

## Discussion

CAKUT represent the primary cause of chronic kidney disease in children and many genes have been attributed to the genesis of this disorder with both dominant and recessive modes of inheritance. CAKUT mainly occur as part of (multi-organ) syndromes but there are also isolated cases described in the literature (1, 2, 10). Just recently, haploinsufficiency of *PBX1* caused by microdeletions was shown to result in bilateral renal hypoplasia and other organ malformations (7). Furthermore, a targeted exome sequencing of 330 genes in 204 unrelated CAKUT patients could identify five novel *de novo* heterozygous loss-of-function variants/deletions in *PBX1* (11).

*PBX1* encodes a transcription factor which promotes protein–protein interaction and is important for organogenesis (12). *Pbx1^−/−^* mice die at an embryonic age and show extensive organ malformations including hypoplastic kidneys with unilateral agenesis (5). In a further publication, *Pbx1*-deficient mice exhibited—besides multiple organ malformations—a pronounced skeletal phenotype with a slender thorax, hunched posture, and axial malformations. *PBX1* is highly expressed in proliferating chondrocytes (6). Additionally, there is strong *PBX1* expression in the fetal brain (7), and it regulates patterning of the cerebral cortex in progenitor neurons in mice (13). To date, two publications reported CAKUT phenotypes related to pathogenic *PBX1* variants/microdeletions; however, only two of the eight patients published by Le Tanno et al. had heterozygous microdeletions only encompassing *PBX1* (7). The phenotype of these patients involved, among other things, bilateral renal hypoplasia with hyperechogenic parenchyma, cryptorchidism, skeletal malformations, and developmental delay. The other patients in this publication had larger deletions involving a more extensive set of genes contributing to the phenotype. The five patients with novel loss-of-function variants/deletions in *PBX1* identified in a targeted exome sequencing study (11) lacked a detailed genotype–phenotype correlation, as only limited information on the extrarenal phenotype was provided. See Table 1 for a detailed summary of the genetic changes in *PBX1* and clinical features described so far.

**Table 1:** Summary of genetic changes in *PBX1* and clinical features reported so far.

Patient	Kidney/urinary tract phenotype	Kidney function	Extrarenal phenotype	Genetic change	Protein change	Mode of inheritance
This report	Bilateral dysplasia, hyperechogenicity	eGFR = 59–90 mL/min/1.73 m^2^	Bilateral cryptorchidism, hypoplastic clavicles, postnatal growth retardation (height third percentile), global developmental delay (including motor and speech delay), impaired intelligence, dysmorphic features (wide nasal bridge, short neck, bilateral overfolding of the helix, bilateral clinodactyly)	c.[413_419delGGGCAGG];[=]	p.[Gly138Valfs*40];[=]	*de novo*
K175	Bilateral hypoplasia	eGFR = 40 mL/min/1.73 m^2^ (at 21 years)	Deafness, scoliosis	c.[428delA];[=]	p.[Asn143Thrfs*37];[=]	*de novo*
K179	Bilateral cystic hypodysplasia	eGFR = 51 mL/min/1.73 m^2^ (at 11 years)	Dysmorphic features, developmental delay	c.[550C>T];[=]	p.[Arg184*];[=]	*de novo*
K186	Bilateral hypoplasia with oligonephronia	Oligohydramnios	No	c.[511-2A>G];[=] (exon 4, splice acceptor site variant)		*de novo*
K181	Hypoplastic horseshoe kidney, absence of corticomedullar differentiation	eGFR = 40 mL/min/1.73 m^2^ (at 39 years)	Deafness	2.5-Mb deletion (encompassing 8 genes)		*de novo*
K136	Unilateral agenesis/small hyperechogenic kidney	Normal renal function (at 18 months)	Dysmorphic features, impaired intelligence	9.1-Mb deletion (encompassing 131 genes)		*de novo*
PT1	Normal kidney phenotype, bifid right ureter, bilateral pelvis dilatation, bilateral VUR, small urethral valve	Not available	Sacral pit, postnatal growth retardation (weight and height < 3rd percentile), severe global developmental delay, neonatal hypotonia, bilateral perceptive hearing loss, dysmorphic features (low-set ears, anteverted nares, prominent philtrum, thick lips, uvula bifida), seizures in infancy	6.0-Mb deletion (chr1:161650414–167622545, encompassing 36 genes)		*de novo*
PT2	Bilateral renal hypoplasia, nephrocalcinosis	eGFR = 40 mL/min/1.73 m^2^ (at 1.5 months)	VSD, ductus arteriosus, severe kyphoscoliosis, postnatal growth retardation (weight and height < 3rd percentile), microcephaly (<3rd percentile), global developmental delay (including motor delay), deep sound hearing loss, dysmorphic features (antimongoloid eyesplit, frontal bossing, anterior fontanelle closed prematurely, low-set, pointed ears), hypermetropism	9.2-Mb deletion (chr1:162703368–171908659, encompassing 62 genes)		*de novo*
PT3	Bilateral renal hypodysplasia, right renal ectopia, hyperechogenicity, dedifferentiation	eGFR = 66 mL/min/1.73 m^2^ (at 5 years)	VSD, ductus arteriosus, sacral pit, mild global developmental delay (including motor and speech delay), dysmorphic features (hypoplastic lobes, left-sided ear pit, long, narrow face, wide nasal bridge, broad nasal tip, mild retrognatia)	2.8-Mb deletion (chr1:163193466 –166058476, encompassing 11 genes)		*de novo*
PT4	Bilateral renal hypoplasia, hyperechogenicity	Normal, eGFR = 106 mL/min/1.73 m^2^ (at 2 years)	Bilateral cryptorchidism, multiple skeletal malformations (shoulder blade, acromioclavicular joint, skull basis, vertebral defects, hip dislocation), postnatal growth retardation (weight 3rd to 10th percentile), motor delay, hypotonia, dysmorphic features (small, low-set, posteriorly rotated ears, abnormal folding of the helix, divergent strabismus, short nose, anteverted nares, prominent philtrum, short neck)	1.5-Mb deletion (chr1:163574086–165092429, only encompassing *PBX1*)		*de novo*
PT5	Bilateral renal hypoplasia	CKD stage 3 (at 3 years)	Corpus callosum hyoplasia, sacral pit, anal malposistion, difficult neonatal adaptation, unilateral vocal fold paralysis, postnatal growth retardation (weight 3rd to 10th, height <3rd percentile), global developmental delay, neonatal hypotonia, hearing loss, dysmorphic features (prominent metopic ridge, broad nasal bridge, abnormally hemmed ears, hypertelorism, epicanthus, divergent strabismus, thin upper lip, long hands and feet, unilateral fifth finger clinodactyly), encopresia, enuresia	3.6-Mb deletion (chr1:163811431–167385298, encompassing 16 genes)		*de novo*
PT6	Bilateral renal hypodysplasia	CKD stage 5 (transplant at 10 months)	Unilateral cryptorchidism, mitral regurgitation, left ventricular hypertrophy, spina bifida occulta, unilateral inguinal hernia, mild global developmental delay (including motor and speech delay), autism spectrum disorder	0.9-Mb deletion (chr1:164330973–165207097, encompassing 2 genes)		unknown
PT7	Horseshoe kidney	Not available	VSD, ASD, ductus arteriosus, cleft of the posterior arch of L5, anal malposition, postnatal growth retardation (weight 3rd, height 3rd—10th percentile), microcephaly (<3rd percentile), global developmental delay (including motor and speech delay), hearing loss, dysmorphic features (thin, low-set hair, anteverted, low-set ears, crumpled, thick helix, thick lower lip, prognathism, mandibular hypermobility, dental malocclusion, bilateral clinodactyly), wide-based gait	6.9-Mb deletion (chr1:164501003–171424595, encompassing 51 genes)		*de novo*
PT8	Bilateral renal hypoplasia, right renal ectopia, right renal dysplasia	Normal, eGFR = 130 mL/min/1.73 m^2^ (at 4.2 years)	Right cryptorchidism, mild global developmental delay (including motor and speech delay), autism spectrum disorder, bilateral conductive hearing loss, dysmorphic features (bilateral ear dysplasia), joint laxity	0.3-Mb deletion (chr1:164523918–164799811, only encompassing *PBX1*)		unknown

*Patients K175, K179, K186, K181, K136 see Ref. (11); patients PT1-8 see Ref. (7). Reference genome for deletion coordinates: hg19; ASD, atrial septal defect; eGFR, estimated glomerular filtration rate; VSD, ventricular septal defects*.

The patient in our report displayed a complex clinical picture with kidney and skeletal malformations and a neuronal phenotype with developmental delay and impairment of intelligence. In addition to the previously published data from *PBX1* mouse models, microdeletions and loss-of-function variants/deletions mentioned above, we provide a detailed description of the phenotype and make the case for an improved diagnostic approach in CAKUT: in this patient our diagnostic algorithm involved SNP array analysis which did not yield a positive result. We then performed WES and identified a novel heterozygous *de novo* 7-bp frameshift deletion in *PBX1* (c.413_419del, p.Gly138Valfs*40). For future CAKUT cases, we recommend directly employing whole-exome or whole-genome sequencing, as these are the apt techniques to identify new pathogenic variants/CNVs in this genetically heterogeneous syndrome. This is especially true for syndromal and familial CAKUT. In patients with isolated CAKUT, however, diagnostic yield is probably rather low, as less than 10% carry variants in 20 known CAKUT genes (2).

## Ethics Statement

This study was carried out in accordance with the recommendations of the Ethics Committee of the Technical University of Munich with written informed consent from all subjects. All subjects gave written informed consent in accordance with the Declaration of Helsinki. The protocol was approved by the Ethics Committee of the Technical University of Munich.

## Author Contributions

KR, MW, TM, and JH were responsible for writing and revision of the manuscript. CS and CM cared for the patient and provided the clinical data. KR, BA, RK-N, and MW did the exome analysis.

## Conflict of Interest Statement

The authors declare that the research was conducted in the absence of any commercial or financial relationships that could be construed as a potential conflict of interest.
